# pH-Dependent Formation of Oriented Zinc Oxide Nanostructures in the Presence of Tannic Acid

**DOI:** 10.3390/nano11010034

**Published:** 2020-12-25

**Authors:** Nurul Akmal Che Lah, Aqilah Kamaruzaman, Sonia Trigueros

**Affiliations:** 1 Faculty of Manufacturing & Mechatronics Engineering Technology, Universiti Malaysia Pahang, Pekan, Pahang 26600, Malaysia; aqilah996@gmail.com; 2 Department of Zoology, University of Oxford, Oxford OX1 3PS, UK; sonia.trigueros@zoo.ox.ac.uk

**Keywords:** ZnO nanostructures, growth mechanism, tannic acid, oriented attachment, Ostwald ripening

## Abstract

To crucially comprehend the relaying factors behind the growth mechanism of ZnO nanostructures, the needs to understand the cause of preferences in the enhancement of desired physicochemical properties are essential. The particular oriented attachment (OA) is believed to become the cause of the classical growth pattern of ZnO nanostructures which is mainly controlled by the Ostwald ripening (OR) process. In the present work, the concerns over the systematic changes in size and the morphological surface of ZnO nanostructures upon exposure to tannic acid (TA) prepared by drop-wise method turns the particles to different surface adjustment state. Here, we assessed the TA capping ability and its tendency to influence the OA process of the ZnO nanostructures. The detailed process of the growth-based TA system via transmission electron microscopy (TEM), scanning electron microscopy (SEM), and FFT autocorrelation revealed the pH effect on their physical properties which proved the transition surface properties state of the particles from rough to smooth states due to oriented attachment. For pure ZnO nanostructures, the surface is almost smooth owing to the strong bonding particles which are then changed to coarsened surface structures upon the introduction of TA. Strong surface adsorption of Zn cations and phenol ligands mediated the agglomerated nanocrystals, surprisingly with smaller nanostructures dimension.

## 1. Introduction

To date, the oriented attachment (OA) or oriented agglomeration growth mechanism developed by Penn and Banfield et al. [1,2,3] has been widely employed to demonstrate the specific assembly of the quantum system into aggregated nanocrystals including the ZnO nanostructures semiconductor. The assembly involves the first-order phase transition from an individual seed crystal of ZnO nucleated upon the exposure of heat that proceeded to the construction of further defect surfaces and the crystal morphology through coalescing, recrystallize, or assemble into larger structures. The occurrence of defect surfaces due to the created edge dislocations that are always observed in most of the secondary nanocrystals (metastable structures) yields the possibility of incorporating the defects into initially defect-free nanoparticles due to the extended lattices through the preferential attachment on specific crystal faces. Generally, this OA crystal growth mechanism is distinct from but fundamentally controls by the classical Ostwald ripening (OR) that provides a route by which distinctive crystal morphologies and nano-architectures can be produced [4,5,6]. These colloidal ZnO nanosheets growth in solution is the result of the combined fundamental effect of OR and the unique OA processes in which the dissolution, precipitation, and ripening during the particle motion, collision, and agglomeration processes formed those unique crystallographic orientations. Hence, the difference is that the formation of complex nanostructure crystals with wide varieties morphologies including rods, chains, multipods, and branched nanowires are mainly explained by OA, which cannot be justified by classical OR [7,8,9].

ZnO is one of the vital materials in semiconductor therefore becomes one of the leading contenders in micro-electric industries materials. The formation of functional surface ZnO nanostructures through OA to construct hierarchically structured ZnO in aid of surfactant has been rapidly growing [10,11]. The unique properties of the ZnO semiconductor are caused by the generation of novel phenomena driven by interactions at their interfaces due to the presence of surfactants in between the aligned nanostructures, that promote the OA based on surfactant interaction-related driving forces for self-assembly formation, kinetic stabilization between the intermediate nanoparticles, and control of coalescence preferential crystal face or the morphology of the nanocrystal [9,12,13]. The hexagonal wurtzite-type structure of ZnO has the $C_{6v}^{2}$ (*P*63*mc*) space group, with two formula units in the unit cell with all atoms occupying $C_{6v}^{2}$ composed of alternating planes of tetrahedral coordinated by O^2−^ and Zn^2+^ ions in an ABAB pattern (hexagonal close packing) at c-axis [14,15]. The produced dipole moment normal to the basal (0001) plane is common for ZnO nanostructures induced by the opposite stack charged ions. This type of nano-assembly could yield better unique materials which give broader application prospects based on their optical, magnetic, electrical, and chemical properties.

The physical OA arrangement of ZnO can be observed by several microscopy techniques. Herein, the main focus of this study is the effect of optimized pH conditions on suspension and dispersion of ZnO nanostructures upon the addition of TA. The dynamic changes in the relationship between particle structure and the agglomeration growth were dictated by analyzing the OA process of crystal growth formation (individually and aggregate clusters). This study focuses on a better understanding of ZnO nanostructures growth from physio-chemical dispersion stability and colloidal suspension morphologies. The results described in this study track the sequence of crystal growth provided as clear evidence that OA rarely operates as the sole crystal growth mechanism and open better understanding of ZnO crystal growth by OA. 

## 2. Materials and Methods 

### 2.1. Materials

ZnO-tannic acid (TA) nanostructures were prepared using the sol-gel method with additional sodium citrate (SC) acting as surface modification agent in an aqueous solution. All chemicals used were of analytical grade and without further purification. Bulk ZnO ($d_{m}\approx$ 50 μm, >99%, Merck, UK) is used as a precursor and SC (C_6_H_5_Na_3_O_7_·2H_2_O, >99%, R&M Chemical, Semenyih, Malaysia) as a stabilizer. TA (C_76_H_52_O_46_) powder (R&M Chemical, Semenyih, Malaysia) is used as surface modification with distilled water (Material Laboratory Faculty of Manufacturing and Mechatronics Engineering Technology, Universiti Malaysia Pahang) is used as a solvent.

### 2.2. Preparation of ZnO-TA Nanostructures 

About 4 mM bulk ZnO (50 μm) is vigorously mixed and added drop-wise with 2 mM SC along with slow stirring for 20 min at 50 °C to obtain a homogenous mixture. When the white suspension is formed we continuously stirred it for a period of 6 h. Stock standard solutions of each chemical reactant are produced straightly by weighing and dilution with ∼90% water to a known volume. The white color sediment formed is centrifuged (12,000 rpm for 12 min) and rinsed a few times with distilled water. The aim is to wash off those impurities that caused improper low stability. The reaction continued for different reaction temperatures (60, 70, 80, and 90 °C) sequentially. To study the effect of TA solution on the synthesized ZnO nanostructures, each concentrated sample is diluted with 50 mL of distilled water, TA was added drop-wise to check its influence on agglomeration formation of ZnO nanostructures. The as-synthesized ZnO nanostructures sample are monitored in comparison with initial concentration-reactant samples (2.5 mM) produced at various temperatures. The added concentrations of TA are 10, 50, 100, and 200 μL.

### 2.3. Characterisation of ZnO-TA Nanostructures

The characterizations of pure ZnO and ZnO-TA nanostructures are carried out using the following instruments. Transmission electron microscopy (TEM-2010, JEOL Ltd., Tokyo, Japan): Grids were examined and all micrographs recorded with an SIS Mega view III camera. The spots were obtained on formvar-coated 200 mesh copper grids (TAAB, Berks, UK) and post-stained with distilled water. Zeiss field emission scanning electron microscopy (FESEM): with an operating voltage at 30 kV equipped with EDX (SIGMA/VP, Darmstadt, Germany) EDS was used to characterize samples morphology.

## 3. Results

### 3.1. The Precipitation of ZnO Nanostructures

The identification of the controlled growth and agglomeration mechanisms of wurtzite ZnO nanocrystals in the presence of TA has been planned based on the implemented experimental work. The primary attention was dedicated to analyzing the initial stages of agglomeration. The hydrothermal reaction between bulk ZnO (precursor) and distilled water (reactant medium) can be expressed based on these equations:

$$\mathrm{ZnO}+H_{2}\to \mathrm{Zn}(\left(\mathrm{OH}{)}_{2}\right)$$

$$\mathrm{Zn}(\left(\mathrm{OH}{)}_{2}\right)+C_{6}H_{5}O_{7}{\mathrm{Na}}_{3}\to C_{6}H_{7}O_{8}{\mathrm{Na}}_{3}+H_{2}O$$

**Figure 1:**
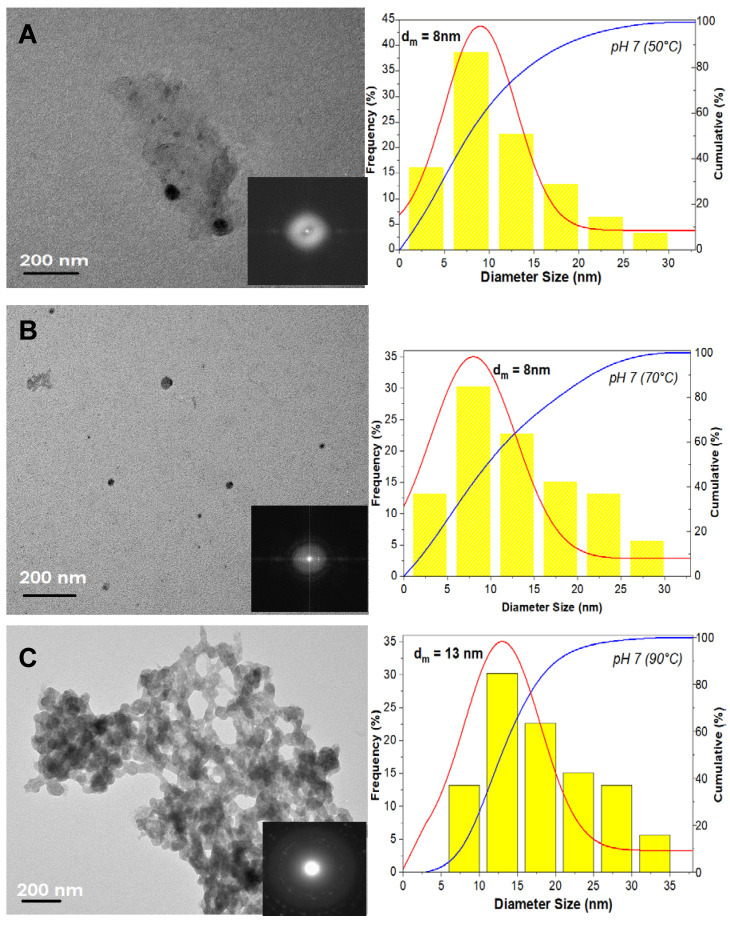

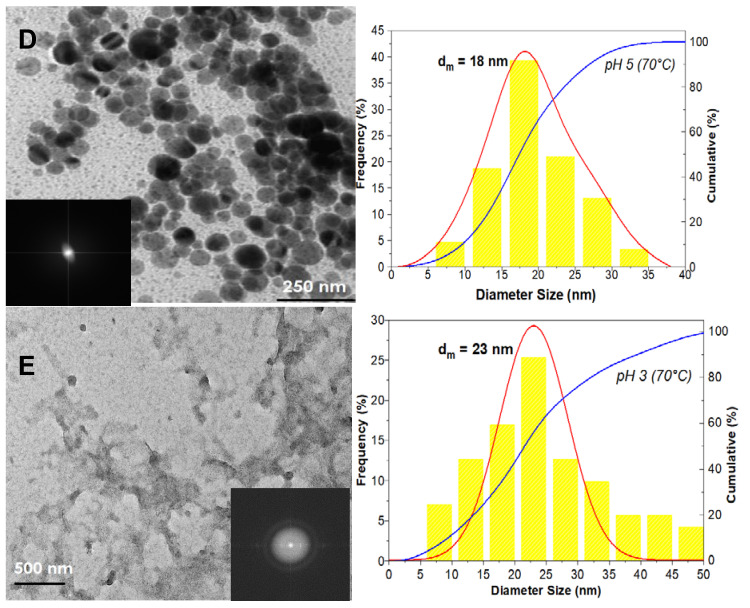
TEM images of pure ZnO and ZnO-TA nanostructures showing particle size distribution plots based on the Gaussian distribution factor (red line) with cumulative analysis (blue line). Pure ZnO nanostructures are synthesized at (**A**) 50, (**B**) 70, and (**C**) 90 °C. Meanwhile, the representative sample of ZnO-TA nanostructures is made using pure ZnO nanostructures synthesized at 70 °C which yield pH value of (**D**) 5 and (**E**) 3. The inset shows the SAED pattern for the respective ZnO samples.

The morphology and particle size distribution of the pure ZnO and ZnO-TA nanostructures where the zero-dimensional structures are visible after a short time of hydrothermal processing is shown in Figure 1. For pure ZnO nanostructures synthesized at 50, 70, and 90 °C (Figure 1A–C), it is proved that the morphological shape of the structures is irregular and polygonal which have polygonal modal mean particle size diameters, $d_{m}$ of about 8 ± 0.2, 8 ± 0.4, and 13 ± 0.5  nm, respectively. During the synthesis of pure ZnO under short processing times and low target temperatures (50 and 70 °C), the particles are not fully evolved. For most of the captured spots, particles are individually assembled and undergo much slower rates of agglomeration, despite their proximity. As small as 5 nm particle diameters have been observed in this study. The formation of this critical size is affected by a range of critical factors including the initial degree of supersaturation, reaction temperature, and pH of the surrounding medium, which are described by classical nucleation theory. The metastable state between the nucleation stage and OR growth stage in a colloidal system is controlled by the thermodynamical growth of OA [16,17]. Initially, these small ZnO nanostructures with negatively charged SC (C_6_H_5_O_7_^3−^ ions) surface surfactants would repel the particles from each other, thus hindering the OR growth from promoting self-orientation of OA growth exclusively. It is believed that the SC monolayer adsorbed on the surface of ZnO nanostructure is composed of dangling di-hydrogen anions (H_2_ Citrate-), and their central carboxylate ions set out to functionalize the surface atoms of Zn. As the synthesis reaction temperature is changed to 90 °C, the coalescence events of small particles are observed producing larger $d_{m}$ of ZnO nanostructures and the particles are more firmly attached. This attachment, reminiscent of twinning, occur at their strong assembly appearance yielding in compacting the bundles of attached polygonal nanostructures. It is noted that the particles undergo a process known as crystal facet matching that creates a twin interface of crystal facets via a transient jump to contact behavior which established the OA process. The particles spontaneously attached to form nano polygonal chains in an oriented direction despite the presence of SC ligands. As the crystal facet produced the twin crystal facet interface, the created connective neck between the particles disappears through a rapid diffusion of surface atoms, yielding the formation of a more massive individual particle.

In the present study, SC shows a vital role in adjusting the morphology and the size of ZnO samples. The well-known process for the morphological and size changes have been reported in several publications [13,18,19]. A similar process is adopted in the present study, but for ZnO particles not Zn ion, to produce the ZnO nanostructures which is affected by the SC. In the presence of SC, the citrate anions adsorb metal surface of Zn in the absence of Zn ion thus forming a relatively stable metal-citrate complex in an acidic condition that activates the generation of a smaller unit of bulk ZnO because of the reaction of Zn((OH)_2_) precipitate and C_6_H_5_O_7_^3−^ ions. It caused the further dissolution of Zn(OH)_2_, and the formation of small ZnO occurred at the same time. The C_6_H_5_O_7_^3−^ ions preferentially adsorb on the zinc (001) basal plane as a capping agent [18] which then suppresses the crystal growth along of the (001) preferential direction to form ZnO nanostructures. Within a short period of reaction time (30 min in our study), these nanostructures aggregate and self-assembled. In my opinion it would be better is you say aggregate or self-assembled, even better just self-assembled into metastable polygonal through the OA for minimizing the total energy of the system.

However, TEM images show that prepared ZnO nanostructures retained the polygonal clusters that are more aggregated for ZnO-TA nanostructures made at pH 5 (Figure 1D) and pH 3 (Figure 1E) with the $d_{m}$ of about 18 ± 1.5 and 23 ± 1.5  nm, respectively. In our opinion the formation of a high surface-free energy created by the ultra-small size distribution ($d_{m}$ ~ 8 nm) of ZnO nanostructures expedited the coalescence of adjacent nanostructures. Furthermore, the presence of TA that creates high, medium acidity (low OH^−^/H^+^ ratio) in between the atomic ZnO nanostructures induced the numerous coordinative unsaturated sites on the ZnO surface facilitating particle coalescence at specific facets to create pre-alignment. This condition implies the consequence of surface ligands introduced in the system during OA, regardless of the ligand types, they bond to the surface of ZnO. Hence, desorption of ligands are appraised as to their surfaces that come into contact. The strong force binding would be one of the fundamental factors for the creation of ZnO nano chains via the OA-based growth mechanism. The selected area electron diffraction (SAED) pattern proves the availability of hexagonal wurtzite crystallites structures owned by ZnO based on the characteristic diffraction of a ring pattern (inset in Figure 1). The brighter ring and more prominent spots showed the existence of some larger crystallites. The observed rings were relatively continuous, which denoted the presence of small nanocrystallites in a random orientation.

**Figure 2:**
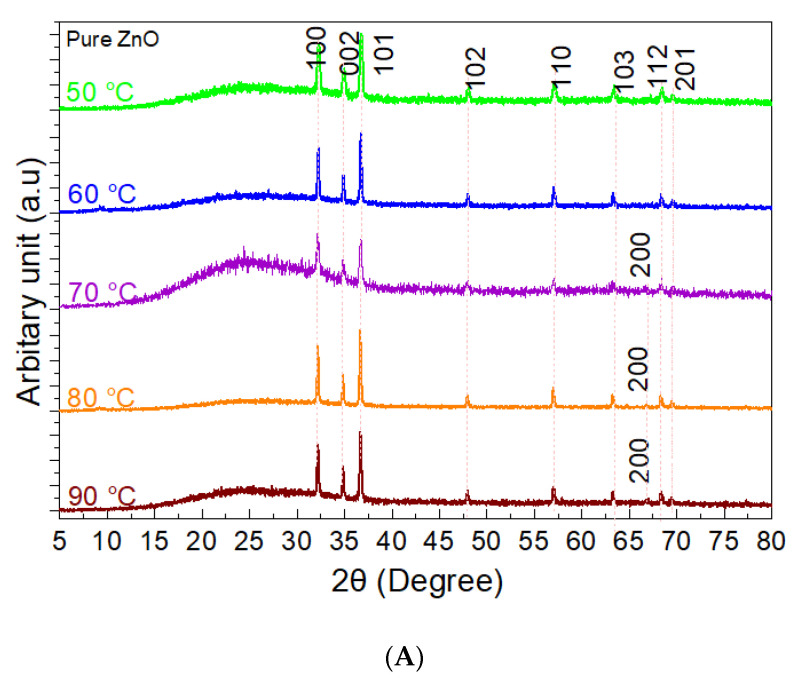

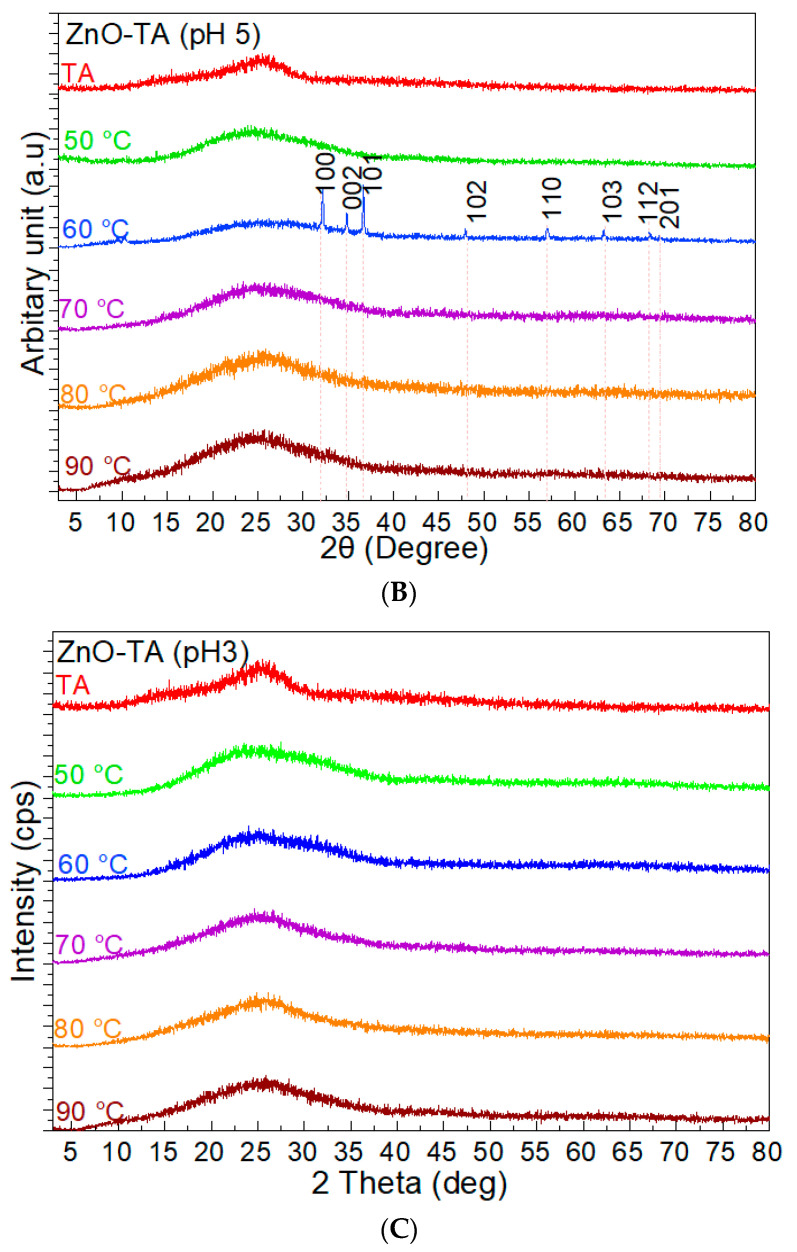
The XRD diffractograms for (**A**) pure ZnO nanostructures synthesized at 50, 60, 70, 80, and 90 °C with (**B**) the XRD peaks of ZnO-TA prepared at pH 5 and (**C**) pH 3 using pure ZnO nanostructures synthesized at 50, 60, 70, 80, and 90 °C. No responses relating to Zn or ZnO have been observed in some of the samples prepared at pH 5 and pH 3, suggesting the domination of TA in all samples.

It is revealed that the polycrystalline hexagonal wurtzite structure of ZnO nanostructures with the observed peak positions in agreement with reported data in Joint Committee on Powder Diffraction Standards (JCPDS, card no: 043-0002 obtained from the library) as indicated in Figure 2. Noted that, for diffractogram test, the amount of sample is expanded for pure ZnO nanostructures with the total of five pure samples synthesized at temperatures of 90, 80, 70, 60, and 50 °C as shown in Figure 2A. Three prominent peaks correspond to reflections from (100), (002), and (101) atomic planes of ZnO phase. It shows the stability, possible directions for grain growth, and are appointed as minimum energy growth phases of ZnO crystal. The presence of other low-intensity reflections corresponds to (102), (110), (103), (200), (112), and (201) atomic planes of hexagonal ZnO lattice. In all the test samples for pure ZnO nanostructures, no peak corresponding to other phases or element/impurity emerged in the XRD analysis. Surprisingly, further added TA concentration in as-synthesized ZnO-TA nanostructures samples inhibits the dominancy of the crystalline phase by vanishing the peak intensities. It is observed that the addition of TA does dispel the diffraction peaks positions of the ZnO nanostructures as demonstrated in Figure 2B,C for samples made at pH 5 and pH 3, respectively, which further implied the absence of any significant lattice on the ZnO wurtzite structure. Only one sample from pH 5 shows the presence of complete ZnO atomic planes. It is believed that during the deposition of the samples on the glass substrate, the amount of ZnO dropped is lower than the TA molecule. Thus, only TA molecules with amorphous spectrum structure in nature are exhibited as they imprinted over ZnO nanostructures in accordance with FESEM analysis.

### 3.2. Assembly of Pure ZnO and ZnO-TA Nanostructures

#### 3.2.1. Influence of the Reaction Temperature

**Figure 3:**
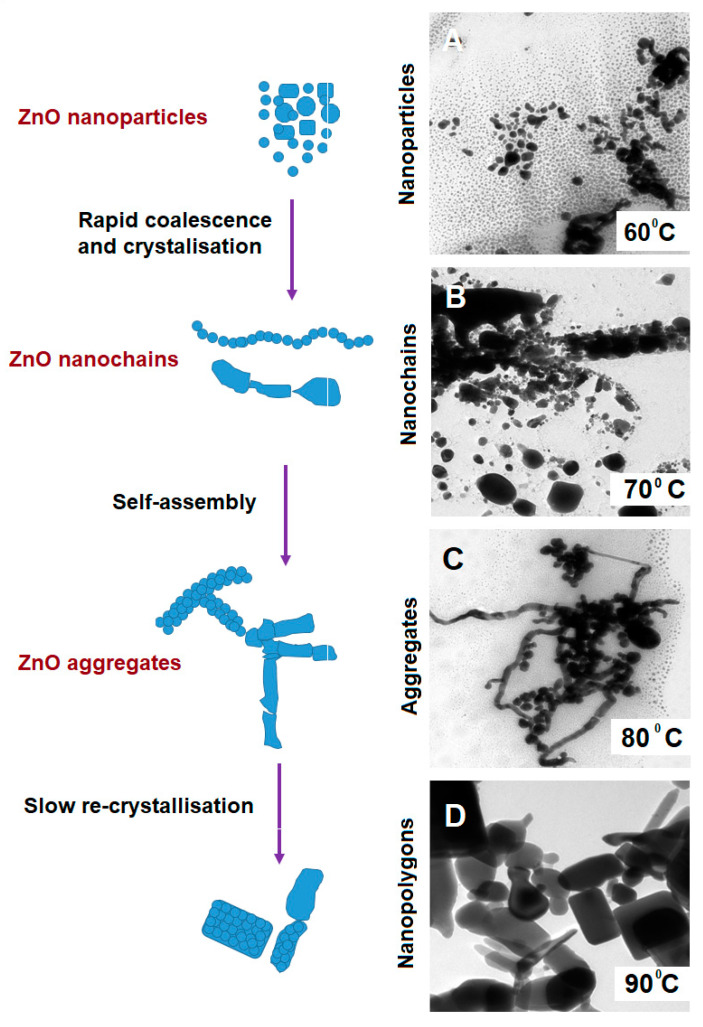
Schematic illustration on the spontaneous growth of ZnO nanoparticles under the governance of the OA mechanism and the corresponding TEM micrographs on ZnO microstructures. Noted that the rapid coalescence occurs as the process changes from (**A**) to (**B**) in the increase of heat. As the process continues, the self assembly occurs (**C**) and further caused slow re-crystallisation as the heat is increased (**D**).

ZnO nanostructures obtained at different reactant temperatures exhibited remarkable activity caused by the active surfaces. Under the setup temperature and indirect generated pressure due to the heat, they automatically underwent similar “nanoparticle-nanochain-agglomerate-polygon array” structural evolution as represented in Figure 3. First, the crystallization-driven self-assembly of the individual tiny ZnO nanoparticles changed into ZnO nano chains. The TEM micrographs demonstrated in Figure 3A–D show the change of ZnO nanostructures assembly due to the influence of reactant temperatures at 60, 70, 80, and 90 °C. The ZnO nano chains that were created in H_2_O, SC, and TA possess irregular curves line, not in straight lines. The ZnO nano chains consist of many small irregularly crystallized ZnO nanoparticles with d_m the range below 20 nm fused together and established a short net-like morphology (Figure 3A). In particular, the ZnO nano chains formed that contained several straight lines that usually created from the round shape of nanoparticles ($d_{m}$ < 10 nm) were connected with each other from different angles. The TEM micrograph in Figure 3B,C showed that the small ZnO nanoparticles coalesced with each other and induced OA process. The typical crystallographic orientation is observed at the boundary among the nanoparticles. This aggregated process exactly represented the common growth mechanism of OA [20]. Similar OA mechanism took place for the ZnO nanoparticles as the temperature increases (Figure 3D). As noted beforehand, OA growth is the intermediate thermodynamically metastable state between the ripening nucleation process stage and OR growth stage in a dispersed medium nanosystem [16,21,22].

At the initial phase, it is proved that the tiny individual ZnO nanoparticles possess strongly charged surface surfactants that tend to impede the OR growth (diffusion-controlled system growth) which exclusively mediate the OA mechanism. Nonetheless, our results showed that the ZnO nanoparticles are attracted to each other to construct oriented nano chains structure despite the presence of capping ligands. It is supposed that the small size nanoparticles with a narrow size distribution of ZnO nanoparticles condense the surface-free energy that activated and accelerated the coalescence between the adjacent nanoparticles. Furthermore, the bonding between the ZnO nanoparticles and the solvent molecules from the surrounding medium is facilitated by the numerous active surface sites The strong interaction forces created between the passivated surface of uncapped ZnO nanoparticles and solvent molecules are strong enough to overcome the different density of nanoparticles and to drive the agglomeration process of ZnO nano chains.

Under static liquid conditions, nanoparticles at close distances tend to agglomerate by moving toward each other, with the facets usually rotating until they achieved their preferred crystal direction alignment, and increased the plan-edge matching for OA determination. Generally, the agglomeration starts with small nanoparticles ($d_{m}$ < 10 nm), loosely packed in a pentagonal configuration under slow rate, despite their proximity. It is noteworthy that, at the area of the high local concentration of nanoparticles, the Brownian diffusivity is decreased [23,24]. Thus, the OA mechanism is sometimes hindered by these nanoparticles pentagonal configuration, which restricts the orientation of the facet alignment with the neighboring particles. Also, the effect of the physical stirring during the synthesis reaction caused the misalignment. Apart from the existence of electrostatic repulsion surrounding the nanoparticles, van der Waals forces are the primary driving force to rapid agglomeration [25,26,27].

The stirring rotation mainly influences the agglomeration speed during the reaction. The dissipated heat energy created by the nanoparticles enables them to overcome the electrostatic kinetic barrier to agglomeration. In static conditions, the binding on the surface limits the rotation. Only when the hydrodynamic forces overcome the binding force, nanoparticles tend to rotate and yield rapid orientation of nano alignment easily. This effect gives significant consequence for very close inter-particle distances, at the order of the particle size. This indicates that hydrodynamic forces play an important role in affecting the motion and alignment of nanoparticles during the stirring period. Thus, induced rapid OA aggregative growth with concurrent surface growth, yield from the multimodal particle size distribution [28,29,30]. The agglomeration may take some time which is estimated to be slower due to the stirring-induced assembly alignment. It is speculated that the primary cause for the OA mechanism is when the combined factors such as the concentration of particles and rapid stirring during mixing, gives significant crystallization pathway. This observation suggested that the OA mechanism occurred in addition to OR, in the presence of slow dissolution and further recrystallisation process.

#### 3.2.2. pH Influence

It has been proposed that the propensity to undergo OA is dependent upon the anisotropy of nanoparticle surface. In this study we show that suspensions with low pH values (3–5) caused those nanostructures edges to decrease through the slight dissociation of phenolic hydrogen groups in the pH range of natural water, lowering Coulombic repulsion and yielding the kinetically favored banded agglomeration around the edges [31,32]. This indicated that the introduction of a higher concentration of TA would lead to smaller nanostructures as the higher concentration of TA leads to the rapid formation of a greater number of nuclei.

**Figure 4:**
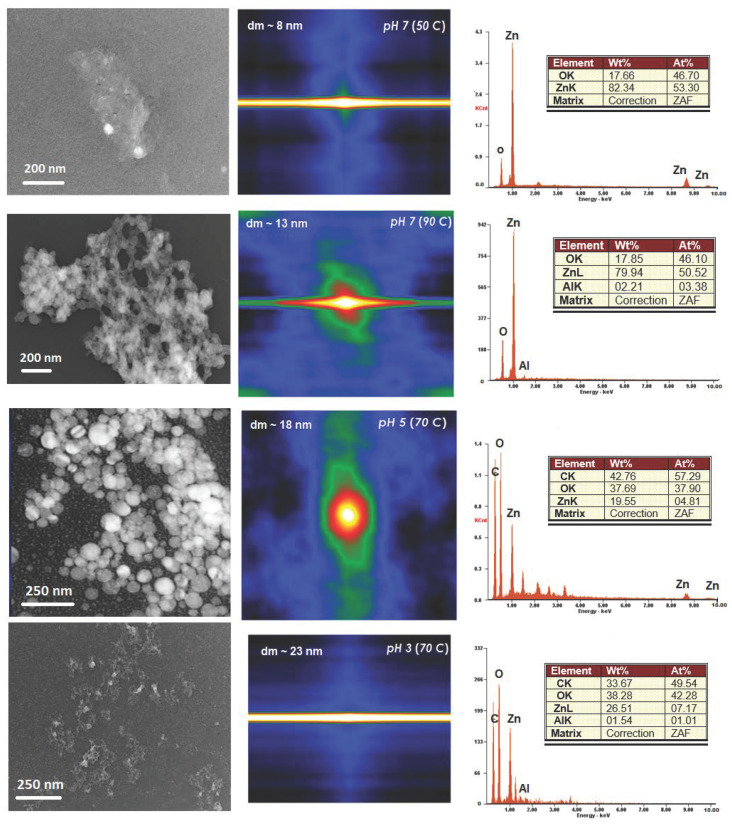
Display of the micrograph TEM images with direct autocorrelation performed on the chosen region.

The adsorbed TA anionic species play a significant role in the OA process mediating the interaction between the acid of the edge phenolic hydrogen group. For instance, phenols are weak acid and lower the pH of the solution., At pH 5, the TA caused an increased precipitation of ZnO nanostructures, as shown in Figure 4. When the pH adjustment was made, the ZnO nanostructures formed with a light brown color suspension. The color of the suspension varied according to the pH value: pH 6, a light brown; pH 5, a pale dark brown; pH 3, a deep dark brown. The deep dark brown suspension formed that consists of ZnO and TA is a consequence of the action of TA. This action is a result of the effect of the colloidal association of the TA and zinc hydroxide molecules. For pH range of 5 to 3, the dark brown precipitation formed when the ZnO was oxidized equitably quickly to zinc hydroxide and the TA was mostly undissociated. It should be noted that only at pH more than 7 the formation of ZnO complexes [33,34] takes place as they are highly oxidized and slowly lost from the solution. As the complexes formed with the oxidized ZnO, the Zn ions are released, and black ZnO tannate is precipitated. Therefore, at pH lower than 3 where no complexes were formed, it is believed that abundance of ZnO had been adjusted to pH to 6 and 7 as explained elsewhere [35,36,37] but not included in the present work.

Notably, the difference between pure ZnO and ZnO-TA nanostructures is the higher rate of agglomeration in acidic medium. Hence, using polymer ligand that causes the acid interaction may induce OA and increase the ratio of basal to edge interaction. At pH 3, all the ZnO was oxidized by the time it contacted with TA. Noted that the rate of oxidation of the ZnO nanostructures upon the introduction of TA is a function of pH and the amount of TA itself. At pH 6 with not sufficient amount of TA, the oxidation rate was slowed and a minor effect was observed because of the reducing power of the TA. As the amount of TA increased, which decreased the pH number, the rate of the oxidation increased as supported by the EDX reports (right) from SEM images.

Noted that the definition of “agglomeration” and “aggregation” is different based on the International Union of Pure and Applied Chemistry (IUPAC) for nanostructures. Aggregation is defined as the joining of nanostructures through a strong interparticle bonding, causing irreversible clustering. Whereas, agglomeration is where the dispersed particles in the liquid are brought together through weak bond interaction which could also result in a phase separation known for its reversibility. Therefore, it is deduced that lowering the pH caused a decrease in the weak interactions (e.g., hydrogen bonding) between the nanostructures. Since there is a direct contact between the TA and ZnO atoms upon the reaction, the TA had a remarkable role in governing the reversible agglomeration and dispersion behavior of the prepared ZnO nanostructures.

According to the results above, it is concluded that the pH shows the pure ZnO nanostructures is dissimilar from that of the ZnO-TA nanostructures and that the TA is the leading cause for the new behavior of the ZnO-TA nanostructures. The TA attaches to the surface of ZnO nanostructures via hydrogen bond interactions due to the abundance of phenolic groups after the exposure of ZnO-TA nanostructures.

### 3.3. Autocorrelation

Direct fast Fourier transform (FFT) autocorrelation started with the analysis of the TEM micrograph images, as shown in Figure 4. The direct autocorrelation function from the micrograph images was obtained from the function tab of autocorrelation using Gatan Suite Software. As the autocorrelation determines the self-similarity of an image, the result always shows a peak at the center (each image point is correlated to itself) and an additional structure that ranges from an amorphous background, for a random distribution of dots, to an ordered peak array, for an ordered lattice of dots. The autocorrelation of the micrograph region indicates a definite bright peak of correlation in the middle that is similar to the respective area from the original micrograph. The condition is always observed for autocorrelation functions as, the midpoint where the image is focused and been shifted either in *x*- or *y*-axis directions.

As observed from the micrographs and autocorrelation images, the sides of the mid contours are believed to represent the preferred distance to the nearest other nanostructural units in the image. Since ZnO are crystalline materials, a long-range order which defines the crystalline in the peaks is consistent with the reported work. The long contours in the *x*-axis line would correspond to the size of the structures visible in the image region. Nonetheless, the areas around the central contour may then indicate the existence of a second layer of nanostructure which is usually partially visible as proved by the TEM micrographs. The FFT autocorrelation method is a rapid technique with less aliasing problem. However, there is an obvious aliasing problem for samples pH 7 (50 °C) and pH 3 (70 °C) that affects the direct FFT correlation method. The effect is observed by the rise in the *x*-axis as a straight line due to the non-crystalline nature of the oxide caused by the discrete in-built function of the FFT method. The autocorrelation function analysis on the ZnO surface provides no evidence of dislocations nor discontinuous coverage. These data suggest that the thin surface layer consists of crystalline, not any sort of carbonaceous materials.

Noted that the irregular shape of the central contours may be correlated to different shapes of structural units exists in each region. Nonetheless, this may also cause by the different structural units for the respective region analyzed. The irregular shape of autocorrelation contour indicated that the *x*-axis is not representative plot of these functions.

## 4. Summary

Theoretically, the OR starts with the initial small crystalline nuclei formation that evolved into a larger particle size to some extent in a supersaturated reaction solution. The OR mechanism is considered as the primary path for crystal growth in chemical reaction systems. Since ZnO nanostructures prepared from the current work does not involve any reducing agent or growth agent, the OR is omitted.

Hence, OA is the primary control of the system nature. In the present work, the OA controls the assembly of the produced ZnO nanostructures through the inevitable repeating attachment events of merged nanostructures that usually occurred on specific lattice-matched crystal facets. The produced output of the current results is not adequate but offers one of the contributed conclusions on the proposed theory so far. The evidence for the corresponding ZnO nanostructures OA formation process becomes evident in which the nanostructure boundary is not an immediate process but instead depicts the assembly arrangement of broad range intermediate nanostructures between nanoparticles and to bulk crystal structures. The formation of intermediate nanostructures which is considered as a bridge for converting small crystals to aggregate crystals plays the primary role in the nanosurfaces changes.

The nanostructures obtained at different synthesis temperatures are considered as adjacent particles in which the tendency for the planes to create integrated structural trends (and not fusion) is higher. Hence, the surface structural changes undergone by the ZnO nanostructures formed at different reactant temperatures became the base reason for the inducement of the crystallization level. The interchange between kinetics (energy motion) and thermodynamics is the primary factor in determining the characteristics of OA. The growth and coarsening of nanostructures started before the OA process. As the OA starts, the smooth surface condition of the nanostructures is achieved. The rough surface of the tiny small nanostructures is controlled by the crystal growth rate that is restrained by diffusion. Typically, such changes in the formation of the nanostructure give a significant effect in inducing suspension characteristics mainly when poorly dispersed nanostructures are formed.

Usually, the fusion structure gives better suspension characteristics of nanostructures due to the excellent stabilization in the liquid medium. However, the OA-grown ZnO nanostructures incline to cluster together, that is supposed to relate to the ability to reduce its overall surface energy by matching crystal lattices and thus decreases the exposed regions and defects. The types of growth processes during the nanostructure formation influence the nanostructure suspension surface.

However, as the TA is introduced into the samples, the acidic medium led to particle crystallite fusion. Based on the thermodynamic and dynamic mechanisms theories, the creation of stable crystalline phases in a solution should be led by the formation of metastable intermediate phases. Theoretically, the rough surface state of the nanostructures upon the TA introduction is caused due to the acidic medium that caused a lower nucleation energy barrier.

The direct autocorrelation using FFT embedded in a Gatan software indicated that the micrographs of the TEM showed the aberrations in the FFT autocorrelation associated with the presence of the aliasing effect. In the *x*-axis of the autocorrelation, the overlap units of the structure caused the x-lineout to dominantly appeared. The autocorrelation function needs to be studied further.

## 5. Conclusions

The present work demonstrated the effect of pH that commonly leads to the OA process in which the surface structure of adjacent ZnO nanostructures tends to attach and create the metastable structures before the size and morphological changes. The TA has a dominant role in the pH-morphological and size-dependent characteristics of the ZnO nanostructures because of the rich phenolic hydrogen group of the ligand which caused the changes in the electron-donating system of the functional group on ZnO nanostructures. Once the alteration occurs, the hydrogen bonding between the ZnO nanostructures induced their agglomeration or dispersion. The correlation function performed shows the mimic condition of the TEM micrographs obtained in this work. It is hoped that the work could be one of the baselines for further exploration on the effect of surface functional group caused by the pH change and stimulates more theoretical and experiments studies related to the ZnO nanostructures.
